# Toward fault tolerant modelling for SCADA based electricity distribution networks, machine learning approach

**DOI:** 10.7717/peerj-cs.554

**Published:** 2021-05-26

**Authors:** Aladdin Masri, Muhannad Al-Jabi

**Affiliations:** Computer Engineering Department, An-Najah National University, Nablus, Palestine

**Keywords:** Internet of things, Machine learning, Power grids, Predictive models, SCADA systems

## Abstract

Maintaining electrical energy is a crucial issue, especially in developing countries with very limited possibilities and recourses. However, the increasing reliance on electrical appliances generates many challenges for operators to fix any fault optimally within minimum time. Even with numerous researches conducted in this area, very few were interested in minimizing the fault duration, especially in the developing countries with very limited resources. Since decision-making requires enough information within minimum time, the integration of information technology with the existing electrical grids is the most appropriate. In this paper, we propose precise and accurate load redistribution estimation models. While several modeling techniques exist, the proposed modeling techniques in this work are based on machine learning models: multiple linear regression, nonlinear regression, and classifier neural network models. The novelty of this work is it introduces a fault-tolerant approach that relies on machine learning and supervisory control and data acquisition system (SCADA). The purpose of this approach is to help electricity distribution companies to maintain power for the customers and to shorten the fault duration from many hours to the minimum possible time. The work was performed based on real data of smart grids split into zones of about 20 transformers. The models’ input data collected from the sensors allocated in the power grid, make the grid becomes able to redistribute the loads by sufficient strategies. To test and validate the models, two powerful modeling tools were used: MATLAB and Anaconda–Python. The results showed an accuracy of about 97% with a standard deviation of 2.3%. The load redistribution was also presented in details. With such eager results, they approve the validity of our model in minimizing the fault duration, by helping the system in taking ideal actions within the optimal time.

## Introduction

Electricity plays a vital role in our everyday life. However, and due to the fast growth of population as well as the widespread of civilization in different geographic locations, electricity distribution faces many challenges. For instance, the current traditional electric grid is more than 50 years old. So, different updates are needed to effectively meet the increasing demands, minimize losses, fault handling, and transport electricity.

Therefore, the engagement of information technology in the electric grid is considered a promising solution to handle many foibles of the traditional electrical grids. As a result, the concept of Smart Grid appeared, which has a lot of interests and motivation. Smart Grids incorporate different effective sensing technologies by employing sensors, communication systems, and control systems into the current electricity grids. Therefore, different improvements are achieved in optimizing assets, operational efficiency, and storage alternatives. Moreover, smart grids integrate a wide range of applications, containing software and hardware technologies that allow services to interface with, and intelligently manage the present electric grid.

One of the major features of a smart grid is the Power Distribution Automation (DA) which means distribution system operation automation, such as functions of SCADA (Zolotova & Landryová, 2000) protection and analogous information technology operations. Distribution automation can mix remote control of switching devices, local automation, and central decision-making into flexible, cohesive, and cost-effective operation architecture for power distribution systems preserving the integrity of the spec.

One of the major problems for the lack of a Distribution Automation system in the developing countries is the investment for creating a communication infrastructure, Remote Terminal Units (RTUs), and installing sensors. Developing countries have many things to do to improve the DA infrastructure as it will be profitable for the downgrading of the Aggregate Technical and Commercial (AT & C) losses (Ghosh, 2012) and providing a better-quality supply.

Cloud Computing (Hayes, 2008) has assisted in delivering IoT to the real world. But not every IoT system can have the leverage of cloud computing. For example, Industrial IoT systems need high-speed control behavior such that, the obtained data from sensors can be instantly processed. The delay caused by cloud in IoT System can produce damage to the systems that need instant processing of the data stream and immediate feedback. Therefore, a new computing approach has appeared called Fog computing (Bonomi et al., 2012).

Such that, it has the essence of cloud computing at the edge, but at the same time relies on the Cloud for an extensive level of historical data processing. However, it forms an overpass between the edge devices and the cloud. Also, Fog computing got much more improvements over cloud computing like less processing delay, reduced latency, and low bandwidth (Tom & Sankaranarayanan, 2017).

Fault location plays a vital role in ensuring the reliability of the electrical power supply. Extensive research work related to fault location algorithms has been carried out over the years, still, accuracy and speed are the major issues (Vilas et al., 2018).

Furthermore, the available real-time data in SCADA databases contain information about operation conditions which is useful for substation maintenance optimization. Such that, substations malfunctioning and failures are monitored via a SCADA system. Consequently, the analyses of these data are useful to evaluate maintenance strategy and plan the reconstructions and replacement of the equipment (Ivanković et al., 2018).

Besides, big data is becoming more and more demanding and critical. Moreover, the supervision and manual decision-making of occurrences of this data is also becoming harder. Nevertheless, the use of SCADA system tends to be more complex, since the decision must be in real-time. One of the motivating solutions for such issues is the use of artificial intelligence or machine learning because of its obliging effects in reducing manual interaction.

Furthermore, to address the automation of power distribution, an integration mechanism has appeared that integrates the Fog Router and Cloud with the IoT SCADA-based system. Each area router is dedicated to a specific region, and it communicates with sensors and controllers in that region and intelligently responds to faults and critical distribution issues. Also, it can communicate with other routers based on predefined protocol (Skripcak & Tanuska, 2013).

In this paper, we present techniques to handle faults in smart grids to minimize the fault duration especially in developing countries, where almost no effort was made previously. Our techniques are based on machine learning, such that our contribution in this work is to make the grid redistribute the load based on different machine learning approaches. Therefore, we built different models, multiple linear regression model, nonlinear regression model, and neural network model. This paper is organized such that ’Literature review’ involves a literature review, ‘System Model’ encloses the system model, ‘System architecture’ presents the system architecture, the components of the system are clarified in ‘System Components’, the description of the methodology is in ‘Methodology’, ‘Results’ contains the produced results and ‘Conclusions’ elaborate the conclusion.

### Literature review

Fault-tolerant power distribution systems attracted increasing attention of the researchers, especially the SCADA-based distribution systems. Tom & Sankaranarayanan (2017) proposed an IoT-based SCADA integrated with Fog for Distribution Automation. The proposed system takes care of the pole transformer health, consumer utilization, outage management, and power quality control. To reduce the latency and internet bandwidth, the system is supported by fog computing that performs real-time streaming analytics.

Skripcak & Tanuska (2013) introduces a prototype design and implementation of a real-time knowledge generation component based on a multiagent approach that can be applied in industrial SCADA systems. Cardwell & Shebanow (2016) discussed the challenges and the efficacy of integrating smart SCADA and smart grid, such that, it discusses the currently used architecture(s) as well as some of the implemented measures to secure those architectures as they grow. Moreover, it considers cutting down the complexity of implementing the many standards afforded by applicable standards and managerial bodies as a means to achieve practical governance. Besides, in Goldenberg & Wool (2013), a model for an intrusion detection system was introduced. The model is specifically designed for Modbus/TCP networks. The presented approach relies on the key deliberation that takes into consideration the highly periodic Modbus traffic from and to a specific PLC. So, the human-machine interface PLC channel of each machine can be modeled using its own unique deterministic finite automaton (DFA). And in Sridhar & Manimaran (2010), cybersecurity attack approaches were extended. The approaches are for the control systems in an electric power system. Such that, after a successful attack on the Automatic Generation Control (AGC) loop, the magnitude of frequency deviation and load-generation imbalance are used to estimate concussion on the physical system. As well, some researchers engaged machine learning approaches in their researches such as Hu (2016) proposed a methodology that clouts automated fault detection systems and controls to be configured through machine learning techniques. The approach relies on merging sensor data and encoding engineering knowledge that is generic to the application system but autonomous of a particular deployment. And Mookiah, Dean & Eberle (2017) presents ongoing security issues related to homes whose power consumption is monitored, regulated, and finally billed to the consumers. Also Zhao et al. (2015) proposed a machine learning model that is based on graphs and using a small amount of training data. The model can detect the fault and determine the possible fault type. And in Chen et al. (2019) developed a novel graph convolutional network (GCN) framework for fault location in power distribution networks. The proposed approach coordinates multiple measurements at different buses while taking system topology into account. The work in Guo et al. (2020) proposed an intelligent, active fault-tolerant system based on a deep neural network. The proposed algorithm used the neural network such that when there is no fault the NN trains each sub-filter. While when a fault occurs, it will predict the fault time data. This data will be used to replace the faulty data into the main filter for fusion. While in Bose (2017), the importance of using artificial intelligence techniques in a smart grid is shown. The paper showed how these techniques provide powerful tools for fault diagnostics and fault-tolerant. It also gave a brief description of several AI-based example applications. Moreover, Li, Ota & Dong (2017) proposed a method to perform accurate clustering on the input data using a deep Convolutional Neural Network (CNN) model. The work discussed electrical load forecasting and how to optimize the distribution of energy resources in the smart grid.

Furthermore, different modeling techniques are used to predict and estimate power distribution. In Wang & Yaz (2016) and Gilbert et al. (2019), nonlinear smart grid models were proposed. The work in Wang & Yaz (2016) is based on a real-time state estimation nonlinear framework for the fault-tolerant in the smart power grid synchronization applications. While in Gilbert et al. (2019) a nonlinear autoregressive prediction model for power generation distribution has also been proposed. On the other hand, another work proposed a baseline estimation method using multiple linear regression Matsukawa et al. (2019). However, in Ahmad & Chen (2018), the work presented a complete comparison of three prediction models employing the machine learning-based models.

Machine learning is also used for prediction and power distribution in smart grids. The work in Syed, Refaat & Abu-Rub (2020) proposes a big data management platform to perform the load predictions in real-time, whereas Hafeez, Alimgeer & Khan (2020) proposed a hybrid electric load forecasting model based on an ANN-based accurate and fast converging (AFC-ANN), and long short-term memory (LSTM). In Vidal, Pozo & Tutivén (2018) a data-driven multi-fault detection and classification strategy is developed. The work aims to detect and classify multiple faults. Also, Lei, Liu & Jiang (2019) presents a deep learning-based model for fault diagnosis. The work takes advantage of RNN and LSTM, to present different deep learning models, including CNN.

Consequently, existing literature lack fault handling through load redistribution automation strategies. Even though, most of the developing countries are still using manual techniques for load redistribution which takes a considerable time to complete the optimal load redistribution task that may extend to several hours. Therefore, the novelty in this research is the use of well-known machine learning models to assist the load redistribution automation strategies in developing countries. Because these countries have very limited recourses including poor infrastructure as well as the timeworn existing electricity grid, there are different issues for the use of manual techniques for load redistribution such as the time it consumes, efforts, and cost.

### System model

Different modeling techniques can be used to model a smart grid. In this paper, three well-known machine learning-based models are applied to test the validity of the load redistribution automation assumption, where the models are: (1) multiple linear regression; (2) nonlinear regression, and (3) Neural network classifier model. Each model is applied separately to arrange the priorities of the backup transformers that can handle the malfunctioning transformer load based on three input variables which are: the free load of each transformer, the oil temperature, and the expected power loss between transformers. The regression-based models are applied in this research because of regression simplicity and because their computations can be performed by the processing resources available in the controllers easily. Therefore, for multiple linear and nonlinear regression modeling, we used the MATLAB tools fitlm and fitnlm respectively. While we used the Python Keras library to build the Neural Network classifier model.

### System architecture

**Figure 1:**
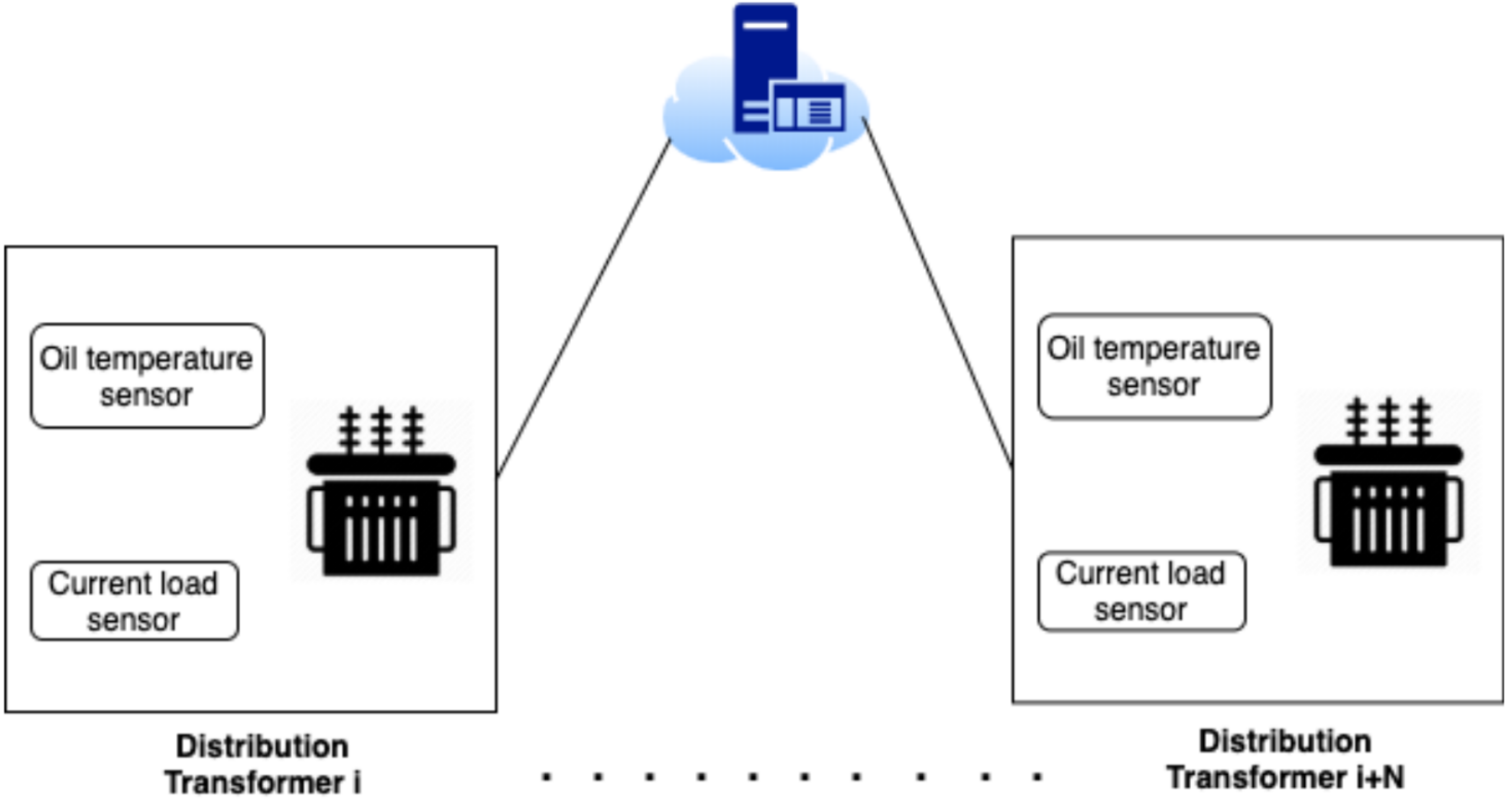
System architecture different zones.

The architecture of the electricity grid in this work can be described as a smart grid where the grid is logically split into different zones where each zone has several transformers and a controller that controls the load distribution among the zone transformers. Such that, each controller is equipped with IoT-based actuators. As shown in Fig. 1, each transformer is equipped with IoT-based measurement sensors, that measure the amount of the current load and the oil temperature. While the power loss between each pair of transformers is estimated by a cloud-based centralized SCADA knowledge system. Transformers within each zone can operate in a fault-tolerant mechanism that can handle the load of the malfunctioning transformer. In other words, when a fault occurs in one transformer, the zone controller will select the most appropriate transformer based on the SCADA machine learning approach.

### System components

**Figure 2:**
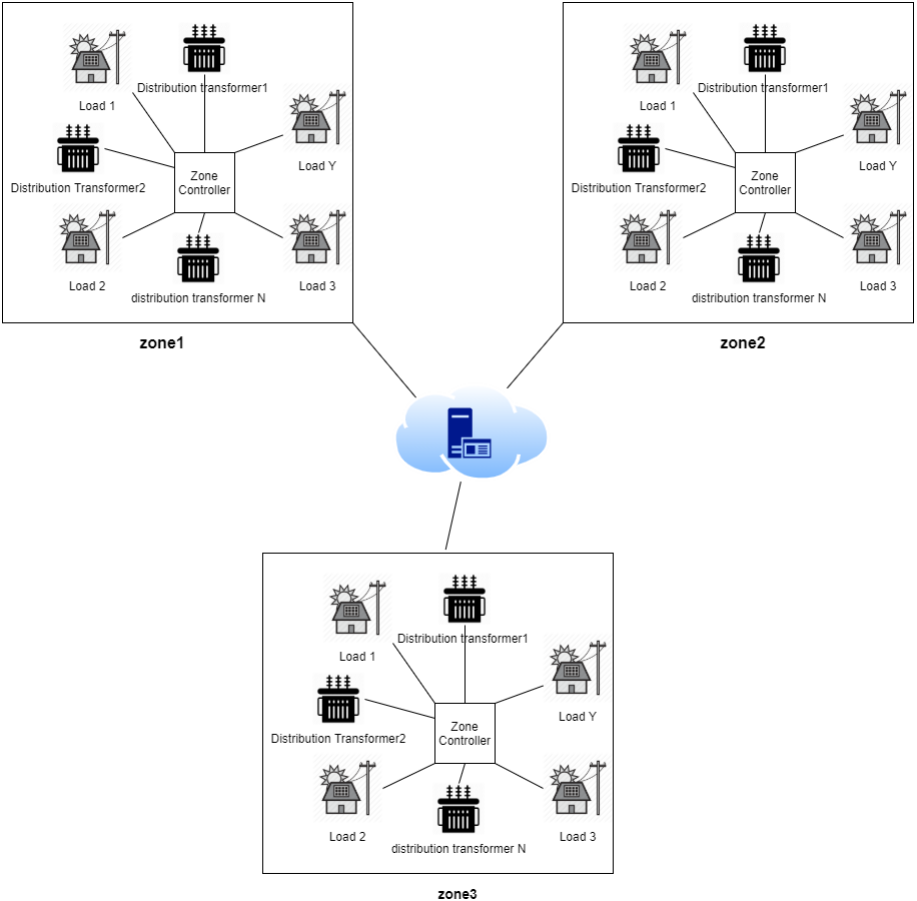
Periodic updates sent by the sensor on each transformer.

Figure 2 provides a general sketch that best describes the smart grid used in our work. such that, the sensors on each transformer periodically send updates to a cloud-based analytics system that, in turn, controls the load distribution mechanism through IoT actuators. So, when a fault is detected in a distribution transformer, the controller allocates the load to the most appropriate transformer according to a prioritization criterion, where the prioritization criterion can be described as follows:

The highest priority is for the Candidate Transformer (CT) that has the highest freeload, the lowest oil temperature, and the lowest estimated energy percentage loss. Hence, the transformers are sorted in a multi-level according to:

(1) Freeload values: from highest to lowest.

(2) Oil temperature values: from lowest to highest.

(3) Estimated energy percentage loss: from lowest to highest.

**Figure 3:**
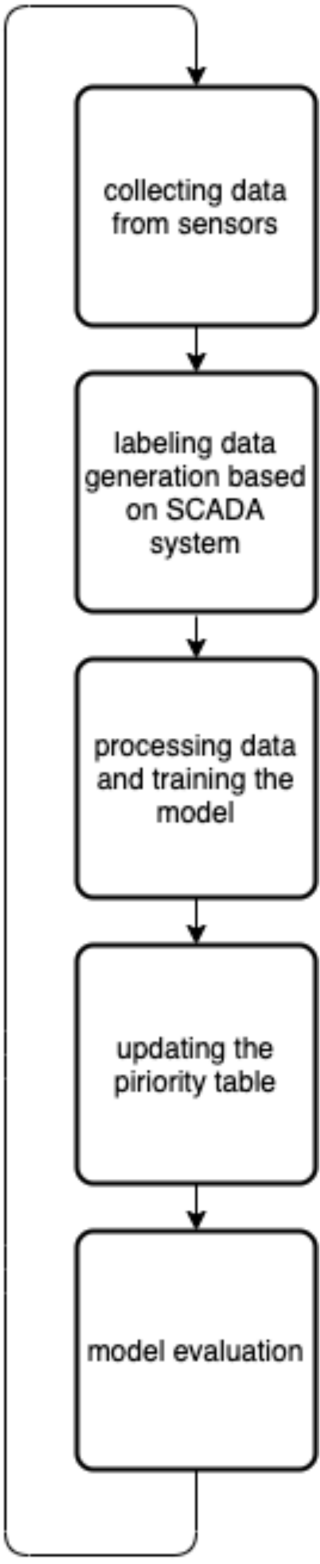
The priority table update algorithm.

As shown in Table 1, the system has a prediction table that has N rows and N columns. Each row contains the priorities of the N-1 candidate transformers (CTs) ordered from highest to lowest with the diagonal element being neglected. Therefore, when the kth transformer fails, it is handled by the ith CT in the Kth row that has the maximum priority value (*P*_*ki*_). Where the prediction table is updated periodically based on an algorithm shown in Fig. 3.

**Table 1:** Priority table.

Distribution Transformer vs Backup Transformer	CT1	CT2	CT3	CTn
S1	X	P12	P13	P1n
S2	P21	X	P23	P2n
S3	P31	P32	X	P3n
Sn	Pn1	Pn2	Pn3	X

## Methodology

In this research, we assumed that the smart grid is SCADA-based, such that the sensors’ readings are collected and monitored through a centralized cloud system. The novelty of this work is by introducing three machine learning models for a load redistribution fault-tolerant mechanism that is integrated with the SCADA-based smart grid. As we aforementioned, each model of the three models was testes separately, where the experimental steps can be described as follows:

(1) The data collection part, was performed as follows:
  (a) For multiple linear regression and nonlinear regression-based models, random readings for a specific zone from the Northern Electricity Distribution Company (NEDCO) were collected to see the accuracy level of building an instantaneous model that can be adapted periodically within short periods.
  (b) Whereas for the neural network model, the readings were collected from the same company for the same zone every six hours for one month.
(2) Models evaluation and verification part, as we mentioned before:

(a) Multiple linear regression and the nonlinear regression-based models were evaluated and verified via the MATLAB tools fitlm and fitnlm respectively.
(b) While the Neural network model is evaluated and verified by the Python Keras library.

Thus, the notations used in these models are:

• *P*_*ki*_: is the ith transformer priority that can handle the load of the kth transformer
• *C*_*i*_: is the ith transformer free capacity; i.e., how much load in KVA can the *CT*_*i*_ handle in addition to its current load.
• *T*_*i*_: *CTi* oil temperature.
• *L*_*i*_: percentage of energy loss during energy transmission along the lines between the kth transformer and the *CT*_*i*_.

since the *P*_*ki*_ value is proportional to *C*_*i*_ and inverse proportional with both *T*_*i*_ and *L*_*i*_, we proposed the model shown in (1)

(1) \begin{eqnarray*}{P}_{ki}= \frac{{C}_{i}^{X}}{{T}_{i}^{Y}\ast {L}_{i}^{Z}} \end{eqnarray*}

where: *X*, *Y*, and *Z* are the regression model parameters.

Thus, these values can be found through different methods and tools:

(1) Multiple linear regression-based model: as shown in Eq. (2), we studied the relation between the natural logarithms of *P*_*ki*_ vs that of *C*_*i*_, *T*_*i*_, and *L*_*i*_. The values of *X*, *Y*, and *Z* can be found through multiple linear regression methods where we used the MATLAB tool fitlm

(2) \begin{eqnarray*}ln({P}_{ki})=Xln({C}_{i})+Yln({T}_{i})+Zln({L}_{i})+W\end{eqnarray*}

where *W* is the intercept

(2) Nonlinear modeling: in this part, we used the same data and the Matlab fitnlm tool to find the *X*, *Y*, and *Z* parameters.

(3) Neural Networks approach: to deal with a highly dynamic environment, it is necessary to consider an approach that is based on neural networks. Such that the system is self-learnable i.e., it can adapt to different numbers of transformers. In this part, we used the Python Keras library to build a neural network that will make the system to select the most appropriate candidate transformer. The wrapper classes provided by the Keras library allow the usage of neural network models developed with Keras in scikit-learn. Also, there is a KerasClassifier class in Keras that can be used as an Estimator in scikit-learn (Pendlebury et al., 2018). The KerasClassifier takes the name of a function as an argument. This function must return the constructed neural network model, ready for training.

In our case, the function creates a baseline neural network for the prioritization classification problem. It creates a simple fully connected network with one hidden layer that contains 45 neurons. The hidden layer uses a rectifier activation function which is a good practice (Schmidt-Hieber, 2020). where the output layer creates 20 output values, one for each class. Note that we used a “softmax” activation function in the output layer (Mohammed & Umaashankar, 2018). This is to ensure the output values are in the range of 0 and 1 and may be used as predicted probabilities. Finally, the network uses the efficient Adam gradient descent optimization algorithm with a logarithmic loss function, which is called “categorical_crossentropy” in Keras (Bock & Weis, 2019; Das, Patra & Mohanty, 2020).

## Results

The dominant limitation in this research is the number of distribution transformers in the zone. Because according to NEDCO, the transformers are grouped into zones based on geographic and topological considerations. And since the limited geographic space in Palestine, each zone contains almost 20 distribution transformers. Therefore, the provided zone from NEDCO to consider in this work contains 20 distribution transformers. Therefore, each transformer is assigned a priority value according to its capability to operate as a backup transformer, where priority values vary from 1 to 20. The distribution transformer which has a priority value of 20 means that this transformer should be considered first to operate as a backup transformer. On the other hand, the transformer has a priority value of 1, which means it should be the last one considered to operate as a backup transformer.

After machine learning the model based on the given assumption, we verified the model in a development machine consists of Intel^®^ Core™ i7-7700HQ CPU @ 2.80 GHz and 16 GB RAM. The classifier model is implemented by using python 3.8 as it is considered to be one of the best machine learning platforms because of its simplicity, flexibility, and its consistency of tremendous libraries for artificial intelligence and machine learning. The Classifier model was built by using different deep learning toolkits such as Numpy, Tensorflow, Keras, Pandas, Matplotlib, and Pylab. While the multiple linear and nonlinear regression models were built by using MATLAB R2016b.

### A. MATLAB regression models

(1) Based on Eq. (2), we performed multiple linear regression using the Matlab fitlm tool. So, the obtained results are shown in Table 2, where the number of observations is 20 and the error degrees of freedom is 16.

**Table 2:** The results of using fitlm on Eq. (2).

Name	Value
W	−2.2957
X	1.0647
Y	−0.0039374
Z	−0.052378

**Figure 4:**
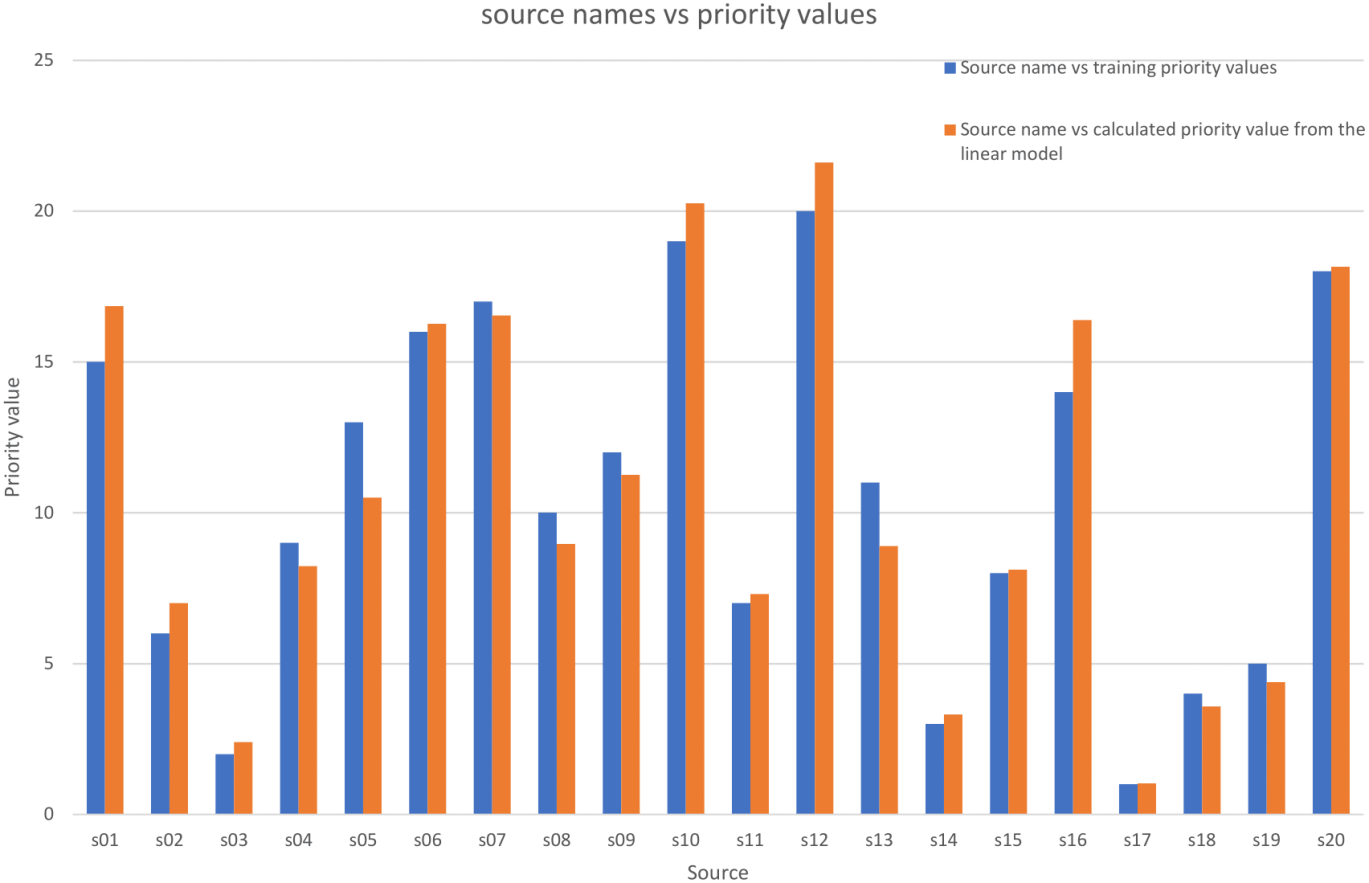
Comparison between the priority training values and the priority calculated values by the multiple linear model.

Figure 4 shows a comparison between the *P*_*ki*_ calculated values using these coefficients and the training values.

**Figure 5:**
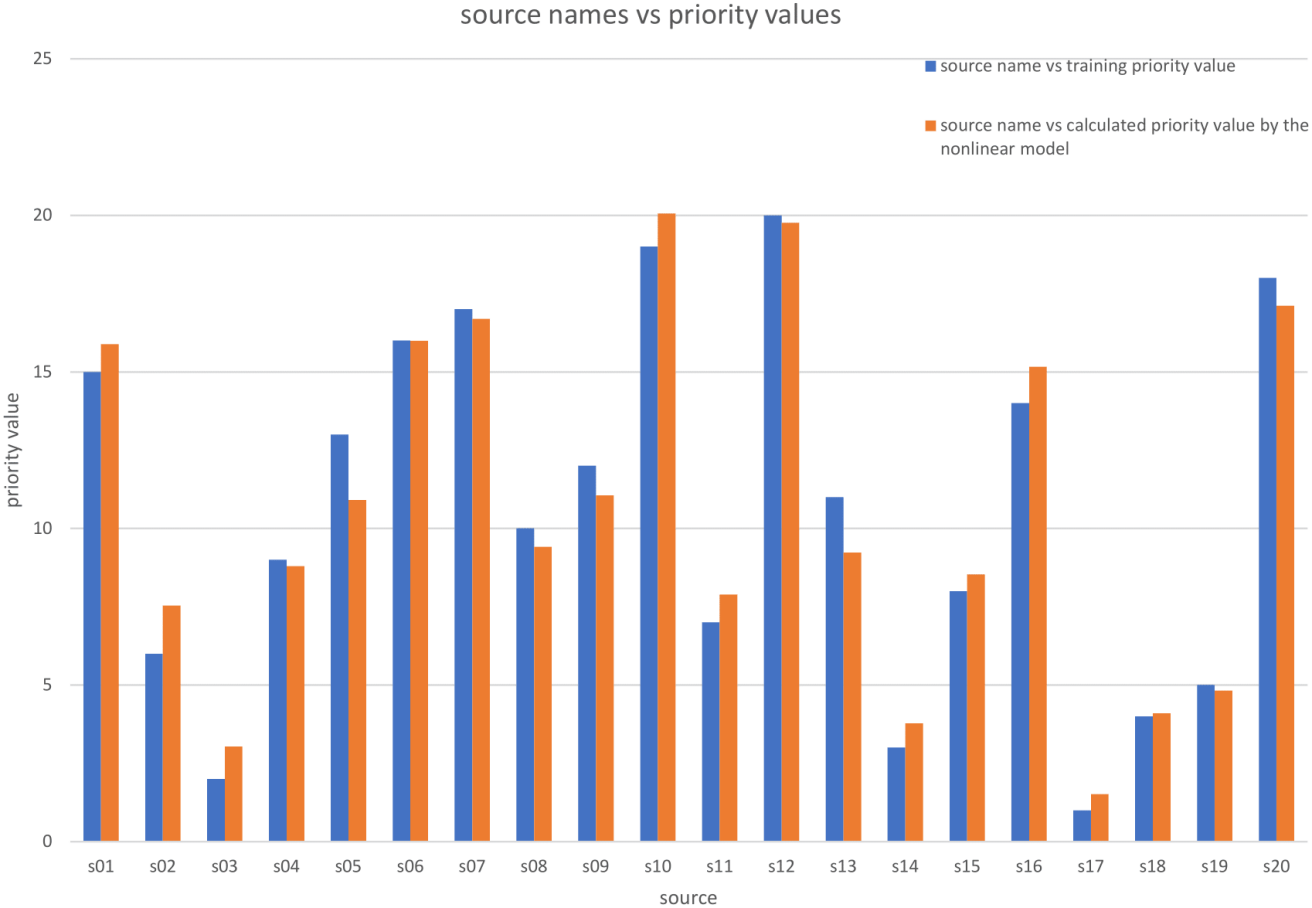
Comparison between the priority training values and the priority calculated values by the nonlinear model.

(2) Based on Eq. (1) we performed nonlinear regression using the Matlab fitnlm tool. The obtained values of the coefficients *X*, *Y*, and *Z* are shown in Table 3:

**Table 3:** The results of using fitnlm on Eq. (1).

Name	Value
*X*	0.82437
*Y*	0.34212
*Z*	0.029426

where the Number of observations is 20 and the Error degrees of freedom is 17. Figure 5 shows a comparison of the *P*_*ki*_ calculated values using these coefficients and the training priority values.

### B. Python neural network model

**Figure 6:**
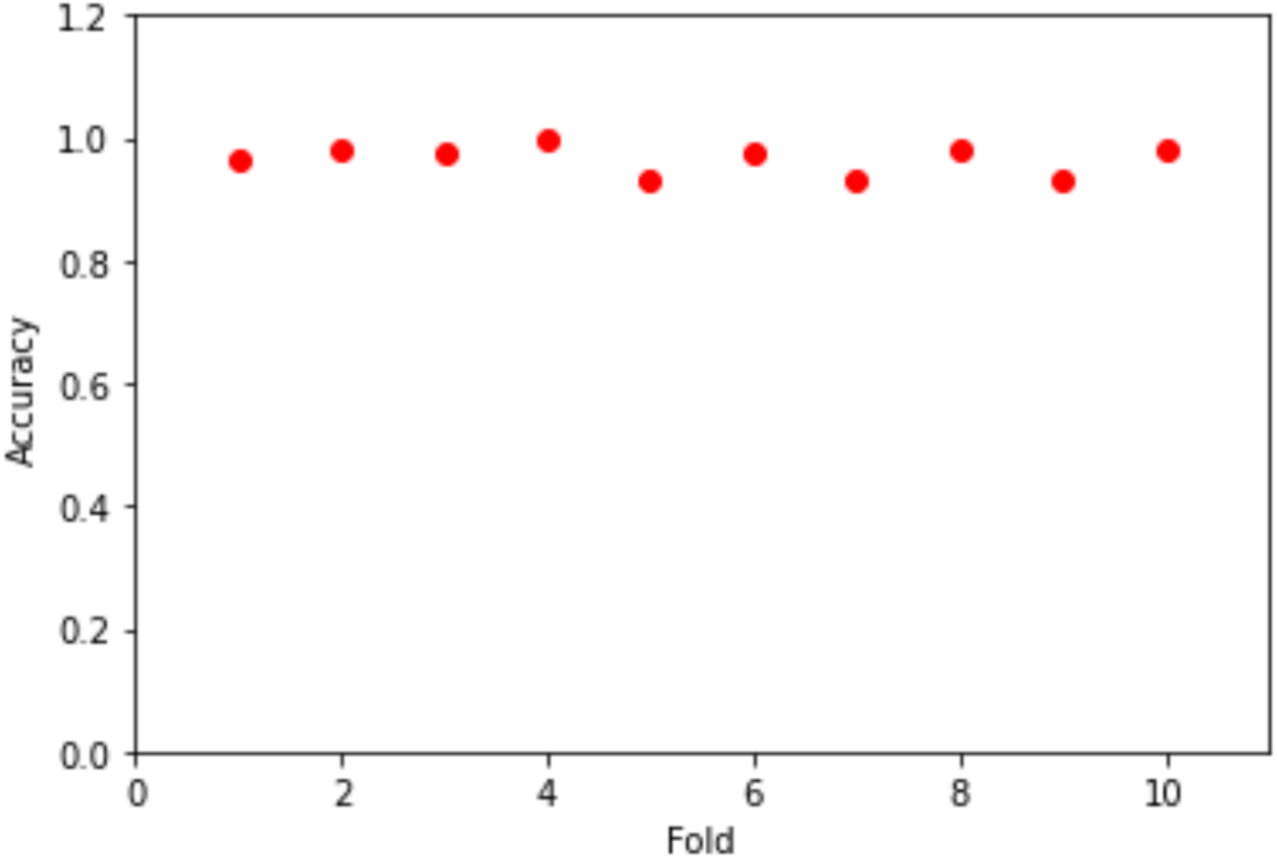
Folds vs accuracy.

To evaluate the neural network model on our training data, the scikit-learn has excellent capability to evaluate models using a suite of techniques. First, we evaluated the model by the KerasClassifier and the k-fold cross-validation using the accuracy metrics as the performance indicator with epochs = 100 and batch_size = 5. Figure 6 shows the folds vs the accuracy. The mean value for the accuracy is 96.58% where the standard deviation is 2.3%.

**Figure 7:**
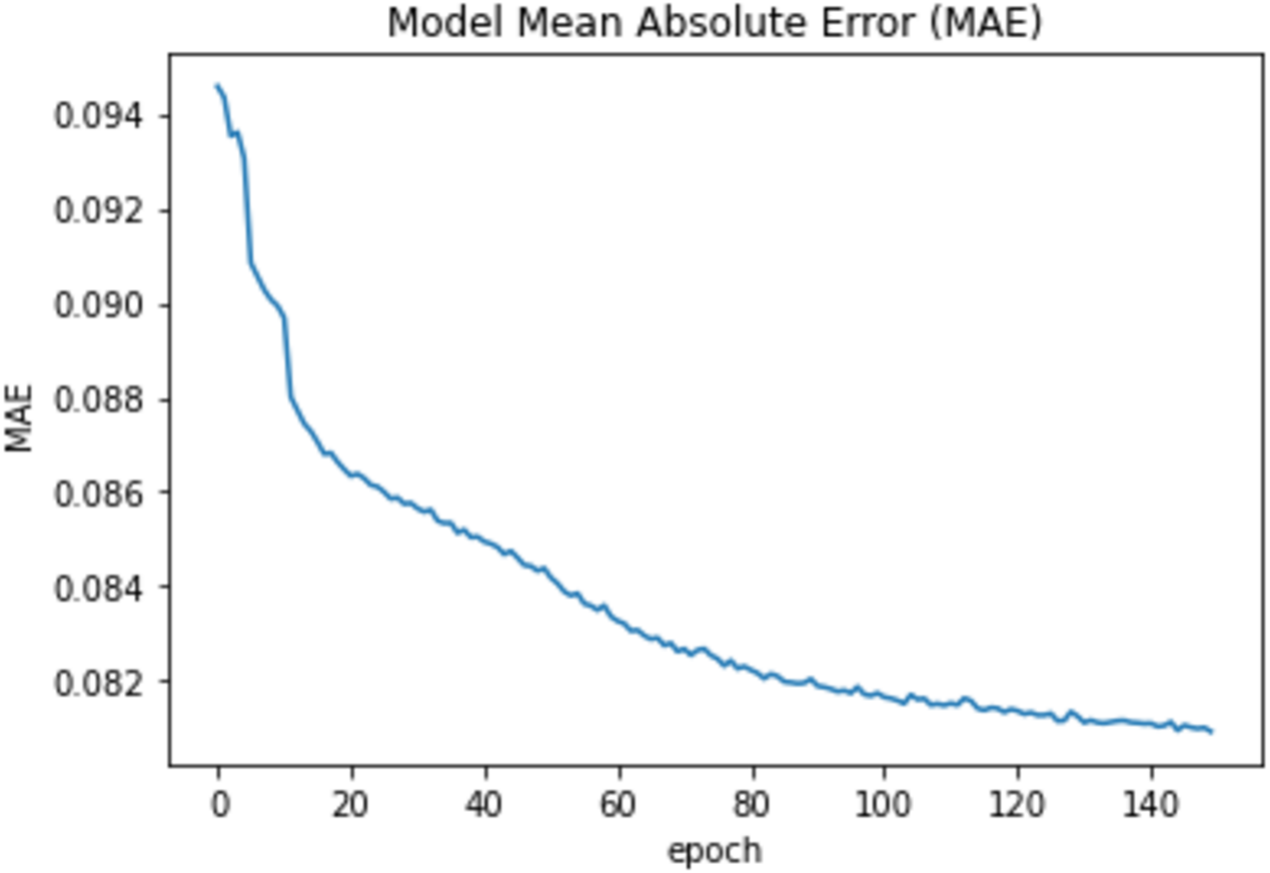
Epochs vs MAE value.

Moreover, we used the fit function with validation_split =0.33 and epochs = 150 and the batch_size = 50 to compare the training accuracy curve with the testing accuracy curve as well as to compare the curves of the training loss and the testing loss. Also, the model was reevaluated using the mean absolute error (MAE). The mean value for the MAE is 0.083 and Fig. 7 shows the epoch vs the MAE value.

### C. models accuracy

**Figure 8:**
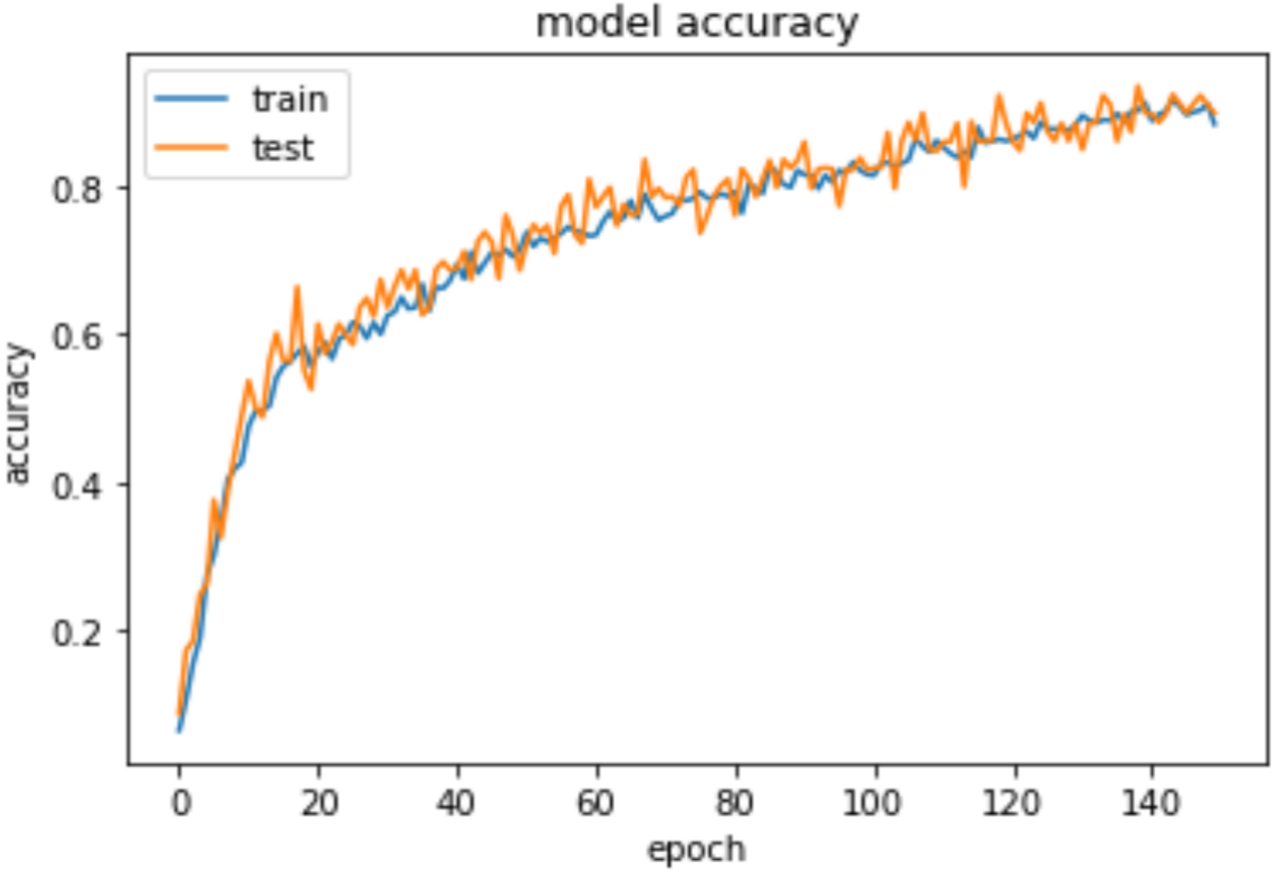
Epochs vs training and testing accuracy.

**Figure 9:**
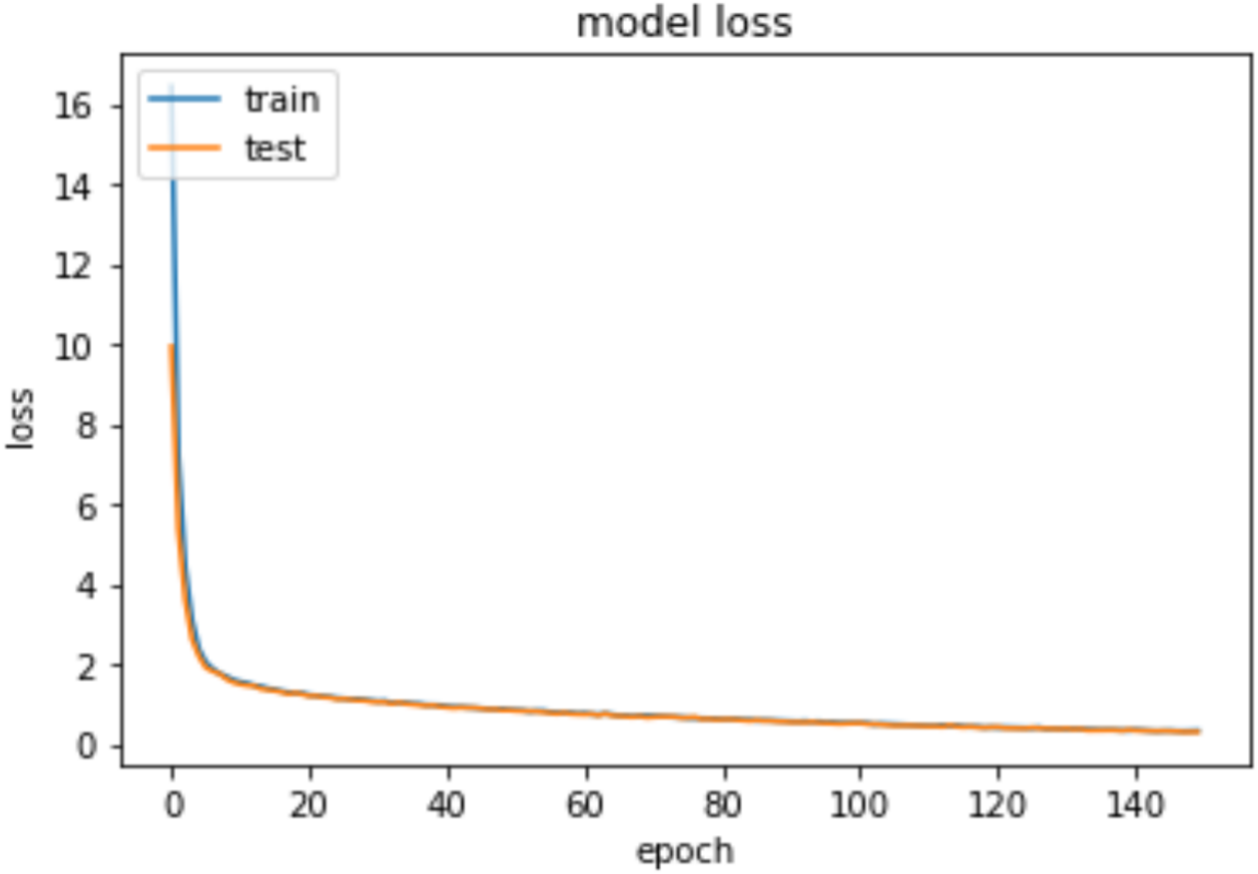
Epochs vs training and testing loss.

To measure the models’ accuracy, the R-Squared and the Adjusted R-Squared are used to measure the model accuracy for both the multiple linear regression and the nonlinear regression models as shown in Table 4. The R-Squared value for the multiple linear regression model is 0.979 and the Adjusted R-Squared value is 0.975. Whereas for the nonlinear regression model, the R-Squared value is 0.972 and the Adjusted R-Squared value is 0.969. Furthermore, Table 4 shows that the accuracy mean value for the Python Neural Network model is 96.58% and the standard deviation is 2.3%, while the mean value for MAE is 0.083. Moreover, Fig. 8 shows that both the training and testing accuracy curves are converging towards 100%. On the other hand, Fig. 9 shows that both the training loss and testing loss converges to 0. Also, Fig. 7 shows that MAE value converges to 0.

**Table 4:** Models accuracy measure values.

Model	Accuracy measure	Value
Multiple linear regression	R-Squared	0.979
	Adjusted R-Squared	0.975
Nonlinear regression	R-Squared	0.972
	Adjusted R-Squared	0.969
Python Neural Network model	accuracy mean value	96.58%
	standard deviation	2.3%
	MAE mean value	0.083

## Conclusions

The durability of electric power is indispensable due to the high dependency on it in our life. However, developing countries suffer from limited resources. Thus, faults occur frequently and last for a protracted time. Thus, accurate load redistribution is vital and the decision-making must be swift. In this paper, we proposed an automatic load redistribution based on different well-known machine learning techniques. The proposed approach aims to:

1- Help in increasing the efficiency of fault-tolerant mechanisms for electricity networks that are relying on the SCADA system and cloud computing
2- Reduce the fault period and increase the reliability of the power grid.

The work has presented three different models: a multiple linear regression model, nonlinear regression model, and neural networks. The models were tested separately and validated on two powerful tools: MATLAB and Python–Keras. Real input data were collected every six hours for a complete month. The results approve the accuracy of our models with an average of 97%. The results also show clearly the proposed techniques are adaptive and scalable. Hence, with the application of any of the proposed techniques in the power grid network, it can help to increase the efficiency and sustainability of electricity in developing countries.

##  Supplemental Information

10.7717/peerj-cs.554/supp-1 Supplemental Information 1 Jupyter file

10.7717/peerj-cs.554/supp-2 Supplemental Information 2 Readme file

10.7717/peerj-cs.554/supp-3 Supplemental Information 3 MAE Jupyter code section

10.7717/peerj-cs.554/supp-4 Supplemental Information 4 Data

## Additional Information and Declarations

### Data Availability

The following information was supplied regarding data availability: The Jupyter files and data are available in the Supplementary Files.
