# Supplementary material for: Toward fault tolerant modelling for SCADA based electricity distribution networks, machine learning approach
Source: PeerJ Comput Sci. 2021 May 26;7:e554. doi: 10.7717/peerj-cs.554 (PMC8176527; doi:10.7717/peerj-cs.554)
Supplement: Supplemental Information 2 [file peerj-cs-07-554-s002.docx]

Uploaded files:

1. scadarandom.ipynb: source code file, Jupyter/Python 3.8
2. scadarandom-mae.ipynb: mae code script
3. scadadata5.xls: contains the data set

column A: Free load

column B: oil temperature

column C: power loss percentage

column D: priority
